# Telomere length regulation through epidermal growth factor receptor signaling in cancer

**DOI:** 10.18632/genesandcancer.140

**Published:** 2017-05

**Authors:** Titto Augustine, Radhashree Maitra, Sanjay Goel

**Affiliations:** ^1^ Albert Einstein College of Medicine and Montefiore Medical Center, Bronx, New York, USA

**Keywords:** telomere length, EGFR pathways/signaling, colorectal cancer, biomarker identification, telomerase

## Abstract

Length of the telomere (TL), a structure at the tip of chromosome that protects and ensures stability, is determined by multi-protein complexes such as telosome/shelterin and telomerase. Earlier studies from our laboratory show that longer TL has potential to be positive predictive biomarker of clinical outcome to anti-epidermal growth factor receptor (EGFR) monoclonal antibody therapy in patients with KRAS WT metastatic colorectal cancer. Although there is extensive literature suggesting the role of shelterin and telomerase, not much literature exists that describes the role of EGFR and KRAS pathway in regulating TL. This detailed review focuses on an insight into various components, including proteins, enzymes and transcription factors, interlinking between EGFR pathways and telomerase that regulate TL.

## INTRODUCTION

Colorectal cancer (CRC) is the third leading cause of cancer-related deaths in the United States when men and women are considered separately, and the second leading cause when both sexes are combined[1, 2]. It is expected to cause about 50,260 deaths during 2017[3]. The majority (close to 70–80%) of CRC are sporadic, while around 20–30% have a hereditary component, due to either uncommon or rare, high-risk, susceptibility syndromes, such as Lynch syndrome (3–4%) and familial adenomatous polyposis (∼1%), or more common but low-risk alleles. A small subset of about 1–2% of CRC cases arises as a consequence of inflammatory bowel diseases. ∼84% of sporadic CRC has genetic instability characterized by chromosomal instability (CIN), whereas as ∼13–16% has hypermutation and show microsatellite instability [4] due to defective DNA mismatch repair (MMR), often associated with wild-type tumor protein p53 (TP53) and a near-diploid pattern of CIN. CpG island methylator phenotype (CIMP) is a feature that induces epigenetic instability by promotor hypermethylation and silencing of a range of tumor suppressor genes, including MLH1, one of the MMR genes[5]. ~3% has ultramutations of DNA polymerase epsilon proofreading [2]. Studies have associated non-hypermutated, microsatellite stable (MSS) CIN with common recurrent mutations in *APC* (81%), *TP53* (60%), *KRAS* (43%), *SMAD4* (10%), *PIK3CA* (18%), *NRAS* (9%) etc. CIN tumors usually arise as a consequence of a combination of oncogene activation (e.g. KRAS, PIK3CA) and tumor suppressor gene inactivation (e.g. APC, SMAD4 and TP53) by allelic loss and mutation, which go along with changes in tumor characteristics in the adenoma to carcinoma sequence[6]. Traditional serrated adenomas, a type of premalignant precursor lesion, frequently (∼80%) have *KRAS* mutations or less often (20–30%) *BRAF* mutations and are MSS or MSI-low[7]. The *KRAS* and *NRAS* mutations are activating oncogenic mutations at codons 12, 13 and 61, and the *BRAF* mutation is the classical V600E activating mutation. Mutations in *KRAS* or *BRAF* lead to hyperactivation of MAP Kinase and PI3K pathways[8].

*RAS* signaling pathway being downstream of EGFR plays a significant role in tumorigenesis. Incorporation of *KRAS* and now the extended *RAS* mutation panel as a predictive marker for anti-EGFR based therapy for CRC is landmark advancement in the pursuit of personalized care for patients with cancer. The median survival of patients with metastatic CRC (mCRC) has improved to 28-30 months mainly due to the availability of newer therapeutic options of EGFR targeted monoclonal antibodies (mAb) such as cetuximab and panitumumab[9]. It is well established that mutations in *RAS* gene predict for lack of response to anti-EGFR mAb. Among the *KRAS* wild type (WT) patients, multiple studies evaluating EGFR based therapy have documented highly variable response rates ranging from 17%–60% that may be because of the presence of other predictive variables that determine responsiveness to EGFR antibodies[10]. Expanding the repertoire of *RAS* mutations to include additional *KRAS* mutations, and *NRAS* and *BRAF* mutations as a screening tool, can further narrow down the spectrum of patients who will benefit from anti-EGFR-based therapy to ~50%[11], requiring further improvements in biomarker discovery and validation.

### Importance of EGFR pathways

The EGFR pathway is stimulated upon ligand binding at the receptor level and subsequent canonical transmission of signals to the nucleus through predominantly three parallel pathways: RAS/RAF/MEK/ERK (MAP Kinase), PI3K/AKT/PTEN/mTOR and JAK/STAT[12]. The role of EGFR pathways in regulating telomerase enzyme is increasingly being recognized[13-15]. To identify additional potential biomarkers of sensitivity to cetuximab and panitumumab, extensive studies have been conducted on these pathways[15-17]. Wormald et al., 2013 has shown that two germline single nucleotide polymorphisms (SNPs) at rs7736074 and rs4975596 located 90 kb upstream of telomerase are associated with somatic mutation of the EGFR pathway and exhibit preliminary prognostic value for response to cetuximab[17]. These variants could potentially contribute to a panel of prognostic biomarkers for assessing whether metastatic CRC patients are likely to derive benefit from cetuximab treatment. By validating using clinical studies, we and others have demonstrated that mutations in the PI3K/AKT/mTOR signaling pathway also predict for resistance to cetuximab[18, 19]. However, this remains clinically investigational because of conflicting data from some other groups[20, 21]. In a continued search for biomarkers, our group has studied telomere length (TL) and its association with sensitivity/resistance to anti-EGFR therapy in CRC[16].

**Figure 1:**
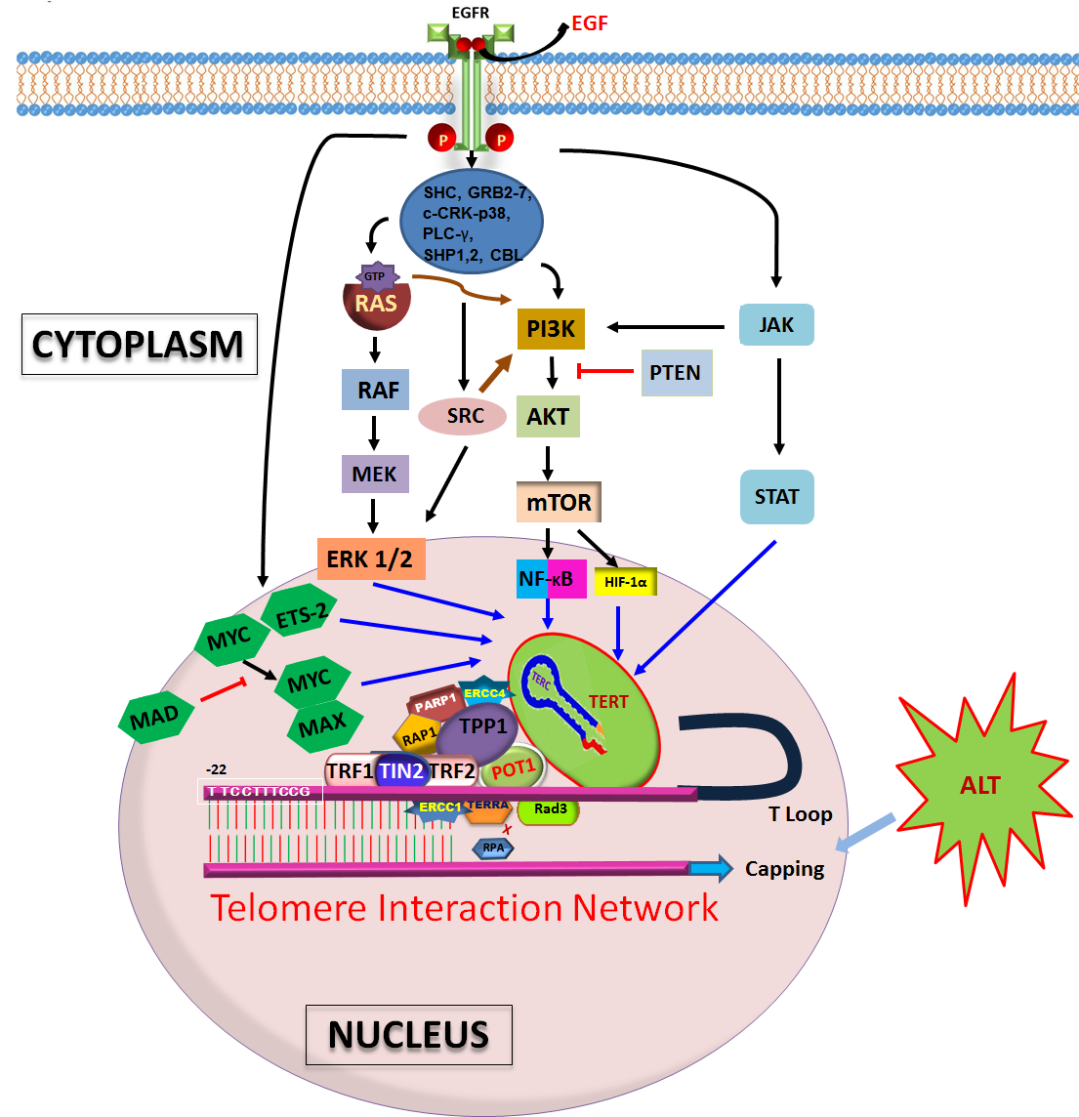
Schematic representation of various components regulating length of telomeres Telomere interaction network comprises telosome/shelterin proteins and telomerase subunits, of which TERT and TERC are prominent. EGF stimulation via EGFR pathways, mainly RAS/RAF/MAPK, PI3K/AKT/MTOR, JAK/STAT and direct activation of transcription factors ETS-2, MYC, MAD and MAX, help to interact with TERT in order to elongate telomeres. ALT, independent of EGFR signaling, is a substitute mechanism for expansion of telomeres.

We reported that TL has potential to be a novel and unique predictive biomarker of clinical outcome (progression free survival/PFS) to anti-EGFR therapy in patients with *KRAS* WT metastatic CRC. There is extensive literature associating EGFR pathway with TL/telomerase. Our studies prove that longer TL corresponds to better therapeutic outcome in patients. Upon comparison, PFSs of other established therapeutic regimens for CRC overlapped when separated into different cohorts based on TL. This helped us to establish a unique interaction between TL and EGFR pathway (Figure 1). An added strength of this study was demonstration of hypothesis in *in vitro* and among patient samples. TL didn't appear to be a prognostic biomarker, but rather, a predictive one. The study, for first time, showed that malignant colonic epithelium corresponding to age-related decline in TL that is widely observed in nonmalignant cells such as peripheral blood leukocytes as well as colonocytes. The study also observed that when patients were categorized as either localized disease (stages 1–3), or stage 4 at diagnosis, there was a statistically significant difference in TL[16]. Cancer tissue in general has lower TL than normal mucosa, and there appears to be a positive correlation between TL and telomerase. Therefore, telomerase, the enzyme constitutively expressed in cancer, is unable to increase TL in tumor cells beyond the adjoining normal mucosa[22].

### Telomere, telomerase and structural complexities

Telomeres are distinctive and repetitive nucleotide sequences found at the ends of linear chromosomes. In vertebrates, they consist of hundreds to thousands of TTAGGG double-stranded DNA tandem repeat sequences and terminate in single-stranded 3’-overhangs (single-stranded DNA/ssDNA) that invade into the double-stranded region to form the so-called telomere loop (T loop) structure[23]. Among its main functions, telomeres mask double strand break DNA damage signals at the extreme of chromosome and prevent chromosomal fusions and thus contribute to genomic stability. Telomeres shorten with each round of cell division and this mechanism limits proliferation of human cells to a finite number of cell divisions by inducing replicative senescence, differentiation, or apoptosis[24]. Telomere shortening can act as a tumor suppressor too[25]. Telomerase, a ribonucleoprotein structure that consists of the reverse transcriptase - telomerase reverse transcriptases (TERT), RNA template - telomerase RNA Component (TERC) and accessory components such as dyskerin (DKC1) - enzyme complex that helps in extension of telomeres, TCAB1 (WRAP53) – localization protein, EST1A (SMG6) – protein aids in telomerase recruitment, and pontin and reptin (RUVBL1 and RUVBL2, respectively). TERT is the key molecule involved in regulation and rate-limiting step in enzymatic activity and carcinogenesis[26]. Telomerase is required for the long-term proliferation of human stem cells and cancer cells. There is mounting evidence for the existence of an important relationship between telomeres and telomerase and cellular aging and cancer[27]. The mechanisms underlying TL maintenance and telomerase expression involve transcriptional, post-transcriptional and epigenetic regulation[15, 28], and an in-depth understanding of these mechanisms may provide novel biomarkers and targets for early detection of disease, determination of disease prognosis, and the development of therapeutics.

A large array of telomeric proteins regulates TL and telomerase activity (TA) and protects telomere ends from being recognized as DNA breaks. These proteins together form the vast network of proteins at the telomeres that ensure genome stability and integrity. The telomere interaction network is anchored by a six protein complex, also called telosome/shelterin, and is composed of telomeric repeat factor 1 (TRF1) and 2 (TRF2), telomeric repeat-binding factor 1 *[TERF1]*-interacting nuclear factor 2 (TIN2), tripeptidyl-peptidase 1 (TPP1), protection of telomere 1 (POT1) and repressor/activator protein 1 (RAP1)[29]. Three of these proteins can directly interact with telomere DNA - TRF1 and TRF2 interact with the telomere duplex region, while POT1 binds to telomere ssDNA. The N-terminal basic domain of TRF2, rich in glycine and arginine residues (GAR or basic domain), can also bind to the non-coding telomeric RNA [30]. Although TPP1 itself has no demonstrable DNA binding activity, it enhances the interaction of POT1 with telomere ssDNA[31]. TPP1 can directly interact with TERT and recruit the telomerase to telomeres[32]. Maintenance of telomeres requires both DNA replication by replication protein A (RPA), which binds to the ssDNA, and telomere ‘capping’ by POT1. Ablation of POT1 leads to aberrant accumulation of RPA at telomeres and activation of the ataxia telangiectasia and Rad3-related kinase [30]-mediated checkpoint response, suggesting that POT1 antagonizes RPA binding to telomeric ssDNA[33]. RAP1 is recruited to telomeres through its interaction with TRF2, and TIN2 is recruited to by binding to TRF1[34, 35]. TIN2 also functions as a bridge connecting TRF1/TRF2 with TPP1[34]. Subunits of shelterin also assemble other interacting proteins on telomeres. They bind to different regions of telomere and induce the formation of a T loop, a cap structure that deters DNA-damage-sensing machinery from mistakenly repairing telomeres. The absence of shelterin causes telomere uncapping and thereby activates damage-signaling pathways that may lead to non-homologous end joining (NHEJ), homology directed repair, senescence, or apoptosis[36].

In addition to telomere DNA and telomeric proteins, another factor in the telomere interaction network is RNA. Telomeres are also constitutively transcribed into telomeric repeat-containing RNAs or TERRA, which are long non-coding RNAs with variable length. TERRA localizes to telomeres and is transcribed from several subtelomeric loci toward chromosome ends[37]. TERRA can interact with a number of telomeric proteins (e.g. TRF1 and TRF2) and has been implicated in telomere heterochromatin maintenance, telomerase regulation and telomere capping. They act as negative regulators of TL based on their ability to inhibit telomerase in vitro[15]. Downregulation of TERRAs occurs during cancer progression, a scenario that requires the efficient elongation of short telomeres by telomerase[38].

Luo et al. in 2015 indicated that some components of telomere interactome can bind to telomere DNA or TERRA (directly or indirectly), suggesting that these proteins may play an essential role in telomere regulation. For example, poly(ADP-ribose)polymerase 1 (PARP1), an interacting protein of RAP1 and telomeric repeat-binding factor 2 (TERF2), functions as regulator of telomere length and end protection, also binds to telomere DNA and TERRA[26]. PARP1 is capable of poly(ADP-ribosyl)ation of TERF2, which affects binding of TERF2 to telomere DNA[39]. Whether PARP1 regulates TERRA stability, localization and/or modification remains to be investigated. Studies identified a RPA-to-POT1 switch on telomeres that is orchestrated by TERRA and heterogeneous nuclear ribonucleoprotein A1 (hnRNPA1). Integrated analysis of different datasets also revealed that core telomeric proteins such as TRF2 can target to gene loci of its interacting partners (e.g. PML), raising the possibility that TRF2 may regulate the dynamics of protein–protein interaction networks and the integrity of telomeres through transcriptional control[40]. TERF2 can recruit excision repair protein ERCC1 along with ERCC4 (ERCC1/XPF complex) heterodimer to the telomeric complex, which helps protect against telomere recombination with interstitial telomere-related sequences, and at the same time prevent NHEJ by blocking its access to the G-strand overhang. TERF2 is also capable of modulating other DNA damage repair (DDR) proteins or complexes such as MRE11A–RAD50–NBN (MRN), which detects presence of uncapped telomeres, Apollo, KU70 and UBR5, which is a chromatin regulator, to telomeres[41, 42]. Interestingly, a number of proteins involved in signal transduction and metabolism appear to interact with core telomeric proteins and/or TERRA, thereby linking metabolic control and specific signaling pathways to telomere maintenance. For example, the subunit of 3-methylcrotonyl-CoA carboxylase (MCCC2) interacts with TRF2 and TERRA. Mutations of MCCC2 have been shown to be associated with the autosomal recessive disorder of leucine catabolism termed 3-MethyIcrotonylglycinuria. The MCCC2–TRF2–TERRA interaction may indicate telomere dysfunction in this metabolic disease[37]. Recently, telomeric proteins have been found to play extra-telomeric roles in biological processes and diseases including NF-κB signaling, obesity regulation, NK cell immunity and neural tumor/stem cell fate control[37].

### Telomere length and EGFR pathways

Immortalization and malignant transformation during cancer involve a complex accumulation of genetic and epigenetic events mainly in the proto-oncogenes and tumor-suppressor genes, as well as the ability to maintain telomeres[14]. TA is observed in almost 90% of human cancers and immortalized cells but not in normal tissues of somatic origin and thus is a critical step for multistep carcinogenesis. In addition to elongating telomeres and deducing an immortalized state, telomerase has other roles in tumor progression[43]. The role of telomere dysfunction in colorectal carcinogenesis is still largely undefined. Several studies demonstrated that telomeres were shorter in CRCs than in adjacent normal mucosa[44-47], but this finding was not confirmed by other studies[48, 49]. Growth signals are directly or indirectly involved in telomerase regulation[28]. EGFR overexpression can lead to malignant transformation and activation of telomerase via survival pathways such as PI3K/AKT/mTOR, MEK/ERK 1/2 and JAK/STAT[50]. Upon activation, various adaptor and effector molecules such as SHC, GRB2-7, c-CRK-p38, PLC-γ, SHP1,2, CBL downstream of EGFR help to link to these pathways[51]. A more thorough understanding of telomerase regulation may provide not only a molecular basis of cancer progression but also as a way to manipulate TA as a potential therapeutic modality. EGF is a representative growth factor that facilitates proliferation of a variety of cell types. Once EGFR-positive cells are exposed to EGF, TA is upregulated following activation of hTERT mRNA expression. This is a rapid effect, observed within 6 hours after treatment. No requirement for de novo protein synthesis was observed, suggesting a direct effect of EGF[43]. There are several lines of evidence that specific signal transduction pathways mediate this regulation. A specific MEK inhibitor of the RAS/MEK/ERK pathway abrogates EGF-induced activation of hTERT. Transactivation of hTERT by EGF requires a specific promoter element (TTCCTTTCCG) located at -22, a consensus binding motif for ETS-2 proteins, known to be the major target of EGFR signaling[43]. Two highly recurrent mutations at two sites within core promoter region of hTERT generate a consensus binding motif for ETS-2, which functions as transcriptional repressor, activator or both to regulate expression of telomerase[15]. Signaling molecules activated by the EGFR, including ERK, SRC, and AKT also have regulatory role on TA. These findings suggest that EGF signals utilize the RAS/MEK/ERK pathway to activate hTERT expression[52].

Another line of study suggests deregulated EGFR pathway induces TA via PI3K/AKT-mediated direct phosphorylation and hypoxia-inducible factor-1α (HIF-1α)-mediated transcriptional regulation of hTERT in cancer. Ionizing radiation increases TA in various cancers by a posttranslational mechanism implicating PI3K/AKT pathway, and is inhibited by PTEN [53, 54]. Yang et al., 2008, suggests hTERT expression is regulated by lysophophatidic acid via PI3K pathway and transcription factor, HIF-1α[53]. Two phosphorylation sites of AKT within hTERT protein help for AKT-dependent phosphorylation and subsequent activation of hTERT. Activated hTERT eventually shuttles from the cytosol to the nucleus[53]. In another study, phosphorylation of hTERT by PKC isoenzymes has been identified as an important mechanism of telomerase regulation[55]. Upregulation of EGFR expression and activation of MAPK signaling pathway were observed in hTERT-immortalized nasopharyngeal epithelial cells. Lack of TA under hypoxic conditions and in the presence of a MAPK kinase 1-specific inhibitor in solid tumors suggests the importance of MAPK pathway in regulating telomerase under hypoxia[56]. The MAPK pathway is involved in the activation of HIF1 expression, and HIF1 has been shown to bind to the promoter region of hTERT, likely at the +1 hypoxia response element site in TERT which is overlapping E-boxes, and regulate TA that was proven by chromatin immunoprecipitation assay and TRAP assay, respectively[57]. HIF-1α binding site within the hTERT core promoter seems to play an important role in the transcriptional regulation or transactivation of hTERT in EGFR overexpressing cells[14]. HIF-1α has been identified as a positive regulator of telomerase expression in different normal and tumor cell lines. Moreover, HIF-1α has been characterized as a downstream target of EGFR and HER2 signaling via the PI3K/AKT pathway and MAPK. Activation and stabilization of HIF-1α are done by phosphorylated AKT and subsequent phosphorylation of HIF-1α protein[50].

### Telomere length, MYC and Wnt signaling

EGFR/MEK/ERK/IKK/mTORC1 is the key upstream pathway of NF-κB activation[58]. Functional NF-κB mediates TA by binding to the κB binding region in the promoter region of hTERT[59]. Constitutive activation of NF-κB signaling also leads to upregulation of other transcription factors such as MYC, MAD and ETS-2, and genes such as IL-6 and BMI-1, and inducing resistance to chemotherapy and radiation and proliferating cancer[60]. In the core promoter-200-bp proximal region-of hTERT, multiple E-boxes are located. MYC binds to these E-boxes through heterodimer formation with MAX proteins and activates transcription of hTERT. This is a direct effect of MYC that does not require *de novo* protein synthesis. MAD proteins are antagonists of MYC and switch from MYC/MAX binding to MAD/MAX binding decreasing promoter activity of hTERT[61]. Overexpression of c-MYC is frequently observed in a wide variety of tumor types, and usually results from chromosome translocation involving the c-MYC genes in addition to gene amplification[62]. It is possible that HIF-1α binding is affected by competition with these factors. Hypoxia downregulates c-MYC expression or facilitates degradation of c-MYC and binding with hTERT[63]. During the competition for hTERT promoter binding between HIF-1α and c-MYC, if there's hypoxia, the promoter is predominantly bound by HIF-1α[57]. Transcriptional derepression of TERT via hyperactivation of MYC might be the rationale for telomere-independent functions of telomerase which include regulation of mitochondrial activity, cell proliferation and apoptosis, WNT/β-catenin signaling, NF-κB signaling, and DDR, all of which may play roles in oncogenesis[64]. MYC-driven oncogenesis is regulated by telomerase. Although overexpression of the c-MYC gene is observed in a significant proportion of tumors, some tumors lack MYC overexpression despite the presence of TA. Upon activation, EGFR drives the phosphorylation/activation of several signal transduction pathways and transcriptionally regulated pathways including NF-κB. Activation of NF-κB promotes tumor progression processes including proliferation, maturation, growth, angiogenesis, invasion, metastasis, clonal expansion and inhibition of apoptosis[65]. RAS/MEK/ERK signaling pathways may be important for hormone/estrogen-mediated transcriptional regulation of hTERT[66]. EGF-activated hTERT expression is mediated via the MEK pathway and transcription factor ETS-2 that targets hTERT promoter in lung cancer cells[13].

MYC and Wnt signaling pathways are key importance for cancer and stem cell biology. TERT is proven to be transcriptionally regulating Wnt–β-catenin signaling pathway and has RNA-dependent RNA polymerase activity when in a complex with RNA component of mitochondrial RNA processing endoribonuclease[67]. β-catenin regulates TERT expression through the interaction with KLF4, a core transcription factor belongs to large SP1-like transcription factor family and important component for pluripotency[68]. TERT functions as a cofactor in β-catenin transcriptional complex through interactions with BRG1 (also known as SMARCA4), which is a SWI/SNF-related chromatin remodeling protein[69]. EGFR-mediated MAPK signaling attenuates Groucho-mediated gene repression, establishing a node for crosstalk between the EGFR, Notch, WNT, and TGF-β signaling pathways[70]. Growth factor and cytokine receptors stimulation induces transcriptional and posttranslational activation of hTERT through JAK/STAT pathway and JAK/PI3K/AKT/HSP90/mTORC1 pathway in hematological malignancies[71, 72].

### Telomere length and cell cycle regulators

Primary/somatic cells divide exponentially making telomeres shorten from ~15 kilobases (kb) until they reach a critical length, 4–6 kb, which induces cell-cycle arrest, monitored by p53 and RB1 and leads to massive genomic instability and cell death via apoptosis or replicative senescence. TA before senescence allows somatic cells to divide indefinitely and maintain a stable genome through a process called immortalization. TA before erosion is complete rescues the genome from instability by re-establishing telomere maintenance. TA after the accumulation of mutations results in an unstable genome, allowing transformed clones that carry multiple mutations to become immortal and oncogenic[73]. Telomere shortening inhibits tumorigenesis in models with intact p53 pathways. EGFR overexpression and mutations in p53 contribute to epithelial to mesenchymal transition (EMT) in TA-immortalized esophageal cells during carcinogenesis[74]. EGFR overexpression triggers oncogene-induced senescence, accompanied by induction of cyclin dependent kinase inhibitors p15INK4B, p16INK4A and p21[75]. EGFR-induced cell cycle progression and proliferation correlated with the phosphorylation and cytoplasmic translocation of p21. On the contrary, overexpression of p21 leads to inhibition of both TA and hTERT mRNA expression in progesterone-positive cells[76]. More than 48 hours of exposure to progesterone promoted cyclin-dependent kinase inhibitor p21/WAF1/CIP1-mediated inhibition of estrogen-induced activation of hTERT mRNA expression[77]. It is unclear how p21 inhibits TA in presence of progesterone, but it is likely attributable to indirect action through cell cycle arrest[28].

### Alternative lengthening of telomeres and EGFR

A subset of cancer/immortalized cells maintain TL for hundreds of population doublings in the absence of TA, and it was therefore deduced that they must have an alternative lengthening of telomeres (ALT) mechanism probably involving genetic [homologous] recombination (HR). Telomerase uses an RNA template for de novo synthesis of telomeric DNA, whereas ALT involves synthesis of new telomeric DNA from a DNA template via HR[78]. The template may be the telomere of another chromosome or another region of the same telomere via t-loop formation or sister telomere recombination[78]. Loss of function or mutations on alpha thalassemia/mental retardation syndrome X-linked (ATRX) or death-domain associated protein (DAXX) genes leads to ALT activation and maintenance, and often associated with growth factor receptor gene amplification in cancers[79, 80]. Nguyen et al. (2013), states that, in brain cancer, ALT phenotype is associated with ATRX mutations and the protein loss is linked with expressions of IDH1 mutant protein and p53, and absence of EGFR amplification[81]. While EGFR genetic polymorphisms being risk factor, DAXX mutations are proved to be limited to only pediatric gliomas[82]. Many molecular details of the ALT mechanism remain unknown, but it has been proposed that various HR proteins are involved.

## SUMMARY

In summary, TL is managed globally by telomerase enzyme, which is a multi-component complex. TL is also controlled by multi-protein shelterin units at their tips. TA occurs through EGFR-mediated pathways and mainly executed by PI3K/AKT/mTOR, RAS/RAF/MEK/MAPK and JAK/STAT signaling. PI3K and MAPK pathways also regulate expression of transcription factor HIF-1α, which is a positive transactivator of hTERT. hTERT core promoter binding is dependent on EGFR-mediated activation of NF-κB through MAPK/mTORC1 pathway and expression of other transcription factors such as MYC, MAD, MAX and ETS-2. MAPK signaling also establishes node for crosstalk between EGFR, Notch, Wnt, and TGF-β signaling pathways. EGFR regulates TA via shortening of TL beyond threshold leading to p53 and p21 induced cell cycle arrest. Homologous recombination based alternative lengthening of telomeres is independent of TA. ALT mechanism is controlled by ATRX and DAXX genes on which EGFR plays a role.
